# Omnidirectional Fingertip Pressure Sensor Using Hall Effect

**DOI:** 10.3390/s21217072

**Published:** 2021-10-25

**Authors:** Moo-Jung Seo, Jae-Chern Yoo

**Affiliations:** Department of Electrical and Computer Engineering, College of Information and Communication Engineering, Sungkyunkwan University, Suwon 440-746, Korea; mtothej92@skku.edu

**Keywords:** omnidirectional, pressure sensor, fingertip, Hall effect, electromagnetic, silicone elastomer

## Abstract

When grasping objects with uneven or varying shapes, accurate pressure measurement on robot fingers is critical for precise robotic gripping operations. However, measuring the pressure from the sides of the fingertips remains challenging owing to the poor omnidirectionality of the pressure sensor. In this study, we propose an omnidirectional sensitive pressure sensor using a cone-shaped magnet slider and Hall sensor embedded in a flexible elastomer, which guarantees taking pressure measurements from any side of the fingertip. The experimental results indicate that the proposed pressure sensor has a high sensitivity (61.34 mV/kPa) in a wide sensing range (4–90 kPa) without blind spots on the fingertip, which shows promising application prospects in robotics.

## 1. Introduction

Sensing the gripping pressure level of objects with uneven or varying shapes is one of the most important technologies in the field of robotic hand applications. Nevertheless, controlling the gripping force has still been challenging [1,2,3,4,5,6,7,8,9], mainly because of a lack of omnidirectionality and sensitivity in fingertip sensors.

Over the past decade, many studies have reported various pressure sensor technologies and transducer methods, most of which are based on one of three sensing mechanisms: piezoresistive [10,11,12], piezoelectric [13,14,15,16,17], and capacitive [18,19,20,21,22,23,24,25,26]. These past studies tried to improve the sensing performance, eventually hoping for being able to delicately regulate the gripping force depending on the fragileness and shapes of objects.

Capacitive pressure sensors are specially designed to have a unique dielectric layer that produces a physical displacement whenever an external force is applied. Then, the physical displacement is expressed as an electrical signal to report its pressure value [24,25,26]. One of the main drawbacks of capacitive transducers is their nonlinear characteristics.

Various studies have proposed methods to improve the sensitivity and non-linearity of pressure sensors. For instance, wrinkly electrodes and pyramidal elastomer di-electric layers have been adopted to help increase the deformation of the elastomer di-electric layers, which improves the sensitivity of the sensors [27,28]. Although this method was somewhat successful in achieving a higher sensitivity, its underlying drawback was that the sensitivity decreased rapidly as the applied pressure increased.

Recently, new methods for manufacturing capacitive sensors have been reported, where novel designs with composite porous elastomers and hydrogel layers were introduced to further improve the sensitivity of capacitive pressure sensors [23,29,30,31]. Nonetheless, their sensitivity remains low, while requiring complicated electrical circuitry.

Piezoresistive sensors convert pressure stimulation into electrical resistance signals directly proportional to the strain caused by the pressure. However, they suffer from being highly sensitive to temperature changes [10,11]. Moreover, there are limitations on scaling down since it can reduce sensitivity [32,33].

To alleviate this issue, several micro-electromechanical system (MEMS) piezoresistive pressure sensors have been proposed [11,34,35]. To improve the sensitivity of the sensor, miniaturized structures, such as a series of rectangular grooves [11], patterned and thinned diaphragm [34], and circular bossed diaphragm with annular grooves [35], were implemented via MEMS fabrication. Despite the improvement in nonlinearity and sensitivity, their use remains limited because of the narrow detection range of pressure and poor overall sensitivity.

Quartz crystals or ceramics have conventionally been used as sensing materials for piezoelectric sensors. These materials generate an electric charge in response to the applied mechanical stress, which is transduced into a measurable electrical quantity proportional to the pressure. Piezoelectric sensors are sensitive to changes in pressure and enable self-powered applications by relying on the charge accumulation during mechanical deformation [15,16,17]. However, the charge generated by piezoelectric sensors decays rapidly, indicating that they cannot be used to measure static pressure; thus, they are not suitable for robotic hand applications.

Besides these sensors, another type of pressure sensor called force-sensing resistor (FSR) has been reported in the literature [36,37,38,39,40,41,42]. The resistance of an FSR sensor decreases with an increase in the force applied to the active sensing surface; thus, it can be used to measure pressure by detecting the applied force. It is well known that FSR sensors offer superior sensitivity over a wide pressure range. The major disadvantage of FSR sensors is their low precision.

There have been almost no research studies on pressure sensors based on the Hall effect. A MEMS Pressure Sensor that has a four-contact Hall structure was introduced in the literature [43] by Hui-Yang Yu, et al. in 2011. It consists of a sealed cavity, a Pyrex glass substrate, and a permanent magnet. The four-contact Hall structure with a rectangular silicon membrane is fabricated to detect the variation of the magnetic field with the deformation of the membrane. However, it is not very useful in robotic applications for sensing pressure from a fingertip. This is because, aside from the problem of bad sensitivity, its lower limit of detection is too high to use as the pressure sensor of a fingertip.

At present, many robotic hand systems are required to handle randomly shaped objects with gripping force feedback. Consequently, they need an omnidirectionally responsive pressure sensor on the fingertip surface with high sensitivity [6,7,8,9]. Such feedback can be sent to the control system to precisely regulate the gripping forces of the fingers. Despite great progress in the sensing mechanism of pressure sensors, realizing a pressure sensor with both high sensitivity and a wide detection range is still an unresolved key issue in robotic hand applications.

There are many difficulties in applying the above-mentioned existing pressure sensors to the robot fingertip’s surface because it is not easy for pressure sensors to simultaneously meet the requirements of omnidirectionality, high sensitivity, and sufficiently wide pressure detection range. Henceforth, there is a significant need to have a dedicated pressure sensor that can measure pressure levels on a finger surface in all directions with high sensitivity over a wide detection range of pressure.

In this study, we propose an omnidirectional-sensitive finger pressure (OFP) sensor using the Hall effect to make a pressure measurement possible from any side of the fingertip with high sensitivity over a wide detection range of pressure. To this end, a cone-shaped magnet slider and Hall sensor are horizontally aligned and integrated inside a flexible elastomer. Moreover, they are assembled and bonded with a 3D printed plastic finger body to form the finger. The magnet slider moves toward the Hall sensor in response to the applied mechanical stress whenever the elastomer is pressed and the Hall sensor detects the strength of the magnetic field emitted from the magnet slider. Owing to the synergistic effect of the magnet slider movement caused by the elastomer deformation from mechanical stress and the Hall sensor detecting the strength of the magnetic field, the presented OFP sensor possesses high sensitivity, a superior wide-detection range, and excellent omnidirectionality.

The rest of the paper is organized as follows:

In Section 2.1, the design and fabrication process of the proposed OFP sensor are explained. Section 2.2 briefly describes the sensing mechanism of the pressure sensor. Section 3.1 compares the performance of the pressure sensor regarding sensitivity and working range with the existing previous works. Section 3.3 explains the experimental results of pressure measurements from various sides of the fingertip. In Section 3.3, the superiority of the proposed OFP sensor is validated in terms of omnidirectionality and sensitivity through an experimental comparison between the OFP and FSR sensors. Finally, the conclusions and future research directions are discussed in Section 4.

## 2. Materials and Methods

**Figure 1:**
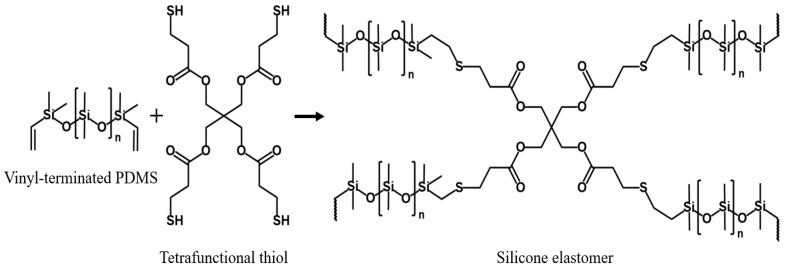
Chemical structure of silicone elastomeric materials used in artificial fingertips, made from vinyl-terminated PDMS(polydimethylsiloxane) and tetrafunctional thiol [47].

The proposed OFP sensor was fabricated by combining a 3D printed finger body (3D Printer: Projet MJP 2500, 3D Systems Inc., Rock Hill, SC, USA) and fingertip to mimic the human finger structure, where the fingertip surface was made of silicone elastomer (Ecoflex 0050, Smooth-On Inc., Macungie, PA, USA), providing a highly elastic soft structure as shown in Figure 1. The Ecoflex 0050 is a material with a Shore hardness = 00−50 and 100% tensile modulus of 83 kPa. Furthermore, it exhibits elastic or skin-like properties [44,45,46] in all directions and thus can imitate realistic human-like fingertip surfaces. In addition, a cone-shaped tunnel was designed inside the silicone elastomer to allow the magnet (Nd-35 with a diameter of 5 mm) slider to move back and forth repeatedly according to the applied pressure, as described in Section 2.1.

### 2.1. Design and Fabrication of the Fingertip Pressure Sensor

**Figure 2:**
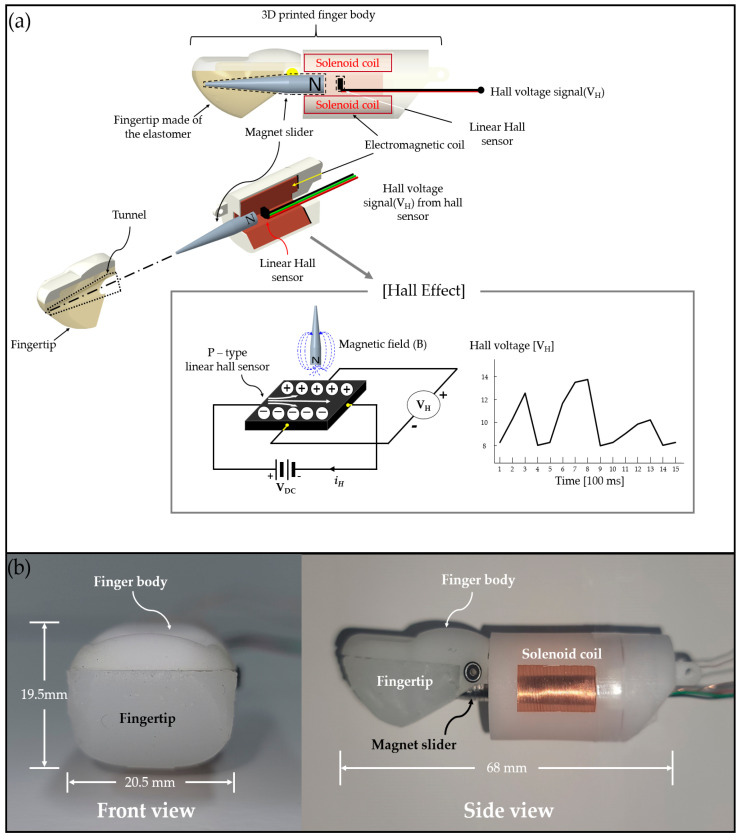
(**a**) Simplified exploded view of the proposed OFP sensor. (**b**) Images showing the fabricated OFP sensor. As shown here, the magnet slider is designed to be cone-shaped with a slippery surface coating. Thus, it can easily move in a horizontal direction on the application of an external force to the fingertip surface. The OFP sensor includes a P-type linear Hall sensor generating a Hall voltage (V_H_), due to the Hall effect [48], activated by a magnetic field from the magnet slider.

The omnidirectional-sensitive fingertip pressure sensor was designed and fabricated according to a human thumb modeled using a 3D computer-aided design (CAD) tool (Creo 7.0). As shown in Figure 2, the fingertip pressure sensor consists of a 3D printed finger body and a hemispheric fingertip made of a soft elastomer with a cone-shaped hollow interior, where they are horizontally aligned and connected to form a moving path (hereinafter, called a tunnel) for the magnet slider. Subsequently, the magnet slider can move smoothly back and forth along the tunnel when an external force is applied to the fingertip surface. To facilitate easy movement of the magnet slider, its surface is coated with an oily grease material made of amine oxide and alpha-olefin.

Because the elastomer of the fingertip is highly cushionable when an external force is applied to its surface, regardless of the applied force direction, it is pressed, and the magnet slider readily moves through the tunnel toward the Hall sensor (SS49E, SEC Electronics Inc., New York, NY, USA), causing V_H_ to increase. In contrast, when the external force is released, the magnet moves back; therefore, V_H_ decreases, while the electromagnet is turned on to help return the magnet slider to its initial position. Therefore, we can figure out how strongly the fingertip surface is pressed by measuring the Hall voltage (V_H_) in real-time.

The Hall sensor is placed at the center of the electromagnet with an air-core solenoid coil, which is arranged orthogonally with respect to the pole of the magnet slider to efficiently detect the magnetic field originating from the magnet slider.

### 2.2. Pressure Sensing Mechanism

This section explains how the OFP sensor is used to measure the pressure from the fingertip surface. We use the Hall effect as a sensing mechanism, which describes how V_H_ changes when the magnet slider moves back and forth along the tunnel.

**Figure 3:**
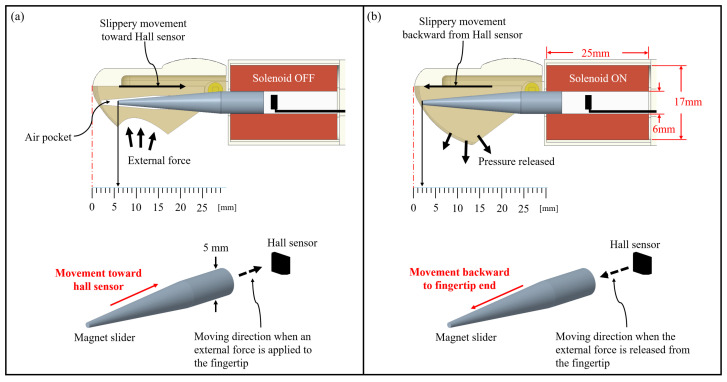
Sensing mechanism of the proposed fingertip pressure sensor based on the Hall effect, where the solenoid has a 6 mm inner and an approximately 17 mm outer diameter with a wire diameter of 0.25 mm. The solenoid is wound 1600 turns per cm. (**a**) When an external force is applied to the fingertip surface, which is a portion of the silicone elastomer, the magnet slider moves toward the Hall sensor, and an air pocket is left behind. (**b**) When the pressure is released, the magnet slider moves away from the Hall sensor.

As illustrated in Figure 3a, when the silicone elastomer portion is pressed by an external force from any side of the fingertip surface, the magnet slider moves horizontally toward the Hall sensor, leading to an increase in V_H_. Figure 3b demonstrates that when the fingertip elastomer portion is free from an external force, the magnet slider moves away from the Hall sensor, which decreases V_H_. Note that the pole of the electromagnetic coil is set to exert a repulsive force against the magnet slider when the external pressure is released. As a result, when the external force is released, the elastomer restores its shape and simultaneously the magnet slider starts to move back to the fingertip. This backward movement is effectively and quickly performed owing to the air pocket, which is a low-pressure area, and repulsive magnetic force between the magnet and electromagnetic coil.

## 3. Experimental Results and Discussion

### 3.1. Performance Comparison of Pressure Sensor Regarding the Sensitivity and Working Range

**Figure 4:**
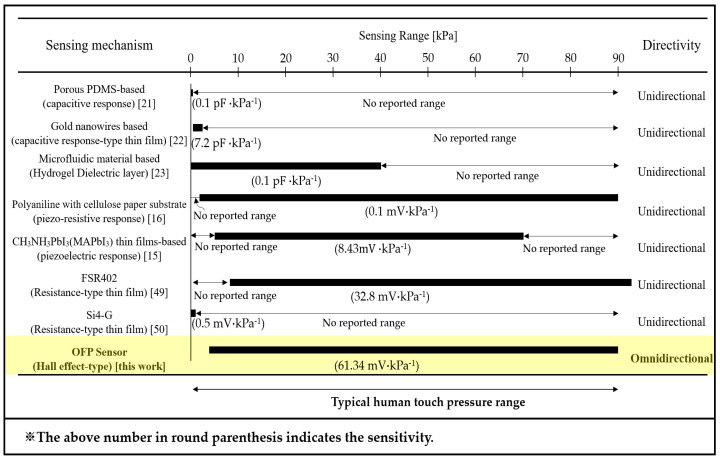
Comparison of the sensitivity and sensing mechanisms of various pressure sensors.

**Figure 5:**
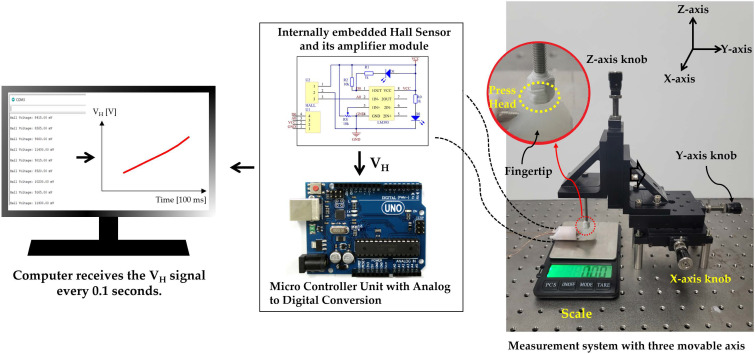
Measurement setup of the OFP sensor using the three-axis moving stage, where the press head contact area with the fingertip surface is $10^{2}{\;\mathrm{mm}}^{2}$.

Figure 4 shows the performance of the proposed fingertip pressure sensor in terms of sensitivity and working range, in which the OFP sensor is compared to various conventional technologies. To this end, the measurement setup shown in Figure 5 was considered to precisely measure the physical quantity pressure applied to the fingertip and convert it into an electrical signal. With this measurement setup, the pressure strength and position of the fingertip can be adjusted using a three-axis moving stage. As stated in Section 2.2, when the fingertip is pressed with a given force, the magnet slider moves toward the Hall sensor. The higher the pressure force, the closer the magnet slider approaches the Hall sensor. The Hall sensor detects the magnetic field radiated from the approaching slider’s magnet using the Hall effect. Therefore, the Hall sensor is a crucial component of the transducer that converts an input mechanical pressure into an electrical output signal (namely, the Hall voltage: V_H_). Furthermore, a high-precision electronic scale (MH-999, MiHee Inc., Gujarat, India) measures the corresponding pressure force in grams (g). Then, the measured Hall voltage is converted into digital data by a microcontroller unit (MCU) and transferred to a computer to be analyzed using MATLAB (The MathWorks, Natick, MA, USA).

As shown in Figure 4, the OFP and FSR402 sensors (Interlink Electronics, Irvine, CA, USA) are the best performing fingertip sensors because they are active over a typical touch pressure range and have high sensitivity over the entire working range. Moreover, apart from the advantage of being an omnidirectional sensitive sensor, our OFP sensor shows superior performance, especially in terms of sensitivity compared to the state-of-the-art touch pressure sensing techniques on robot fingertips. We obtained a sensitivity of 61.34 mV/kPa, which is more competitive than existing studies listed in Figure 4. This is because the magnet slider has a relatively large displacement (approximately 4 mm), yielding a large dynamic range compared to the other sensors with displacements of only a few microns [24,25].

### 3.2. Pressure rueMeasurement from Various Sides of the Fingertip

In this section, to demonstrate that the proposed pressure sensor works as an omnidirectional pressure detector, we measured the variation in V_H_ according to the applied pressure ranging from 4 to 90 kPa from four sides of the fingertip.

**Figure 6:**
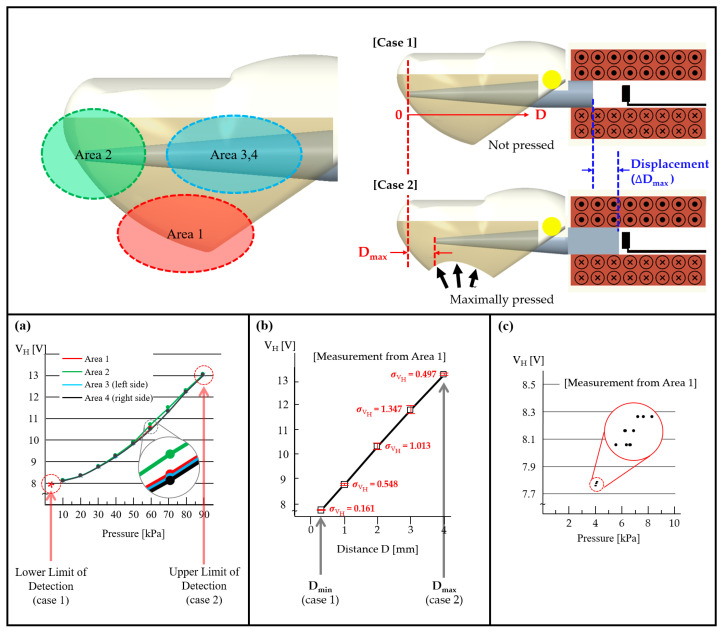
Pressure measurement from four sides of the fingertip. (**a**) Plot of V_H_ versus pressure for different areas. Area 1 is the front portion of the fingertip whereas area 2 is the bottom portion. Areas 3 and 4 indicate the left and right sides of the fingertip, respectively (* denotes the lower limit of detection). (**b**) Plot of V_H_ versus distance D with each tenth trial where error bars indicate the standard deviation. (**c**) Measurement of the lower limit of detection (LLOD) within area 1.

Figure 6a shows how omnidirectional the proposed fingertip for a given pressure level, where the fingertip was made of silicone elastomer which is very soft, very strong, and very “stretchy”, stretching many times its original size without tearing and will rebound to its original form without distortion in all direction [46]. Such excellent stretchable and balloon-like properties lead to a “balloon effect”, i.e., when the squeezed area shrinks, the other part of the elastomer expands, thus minimizing the non-axisymmetric response to the pressure input applied in different directions.

Figure 6a displays the experimental results of the pressure measurement from four sides of the fingertip, where V_H_ versus pressure is plotted with varying pressed positions. These results reveal that our pressure sensor is not only omnidirectional but also has an approximately constant linear relationship between V_H_ and pressure in all pressure directions; thus, the OFP sensor can reliably detect the pressure coming from all directions on the fingertip surface. Case 1 denotes when no external pressure is applied, while case 2 denotes when the external force reaches the maximum value of the detectable range, resulting in a maximal displacement $\left(\Delta D_{\max}\right)$ of approximately 4 mm. In addition, as shown in Figure 6b, we conducted an experiment to examine the V_H_ variation with respect to distance (D), where V_H_ is directly proportional to the displacement D with a relatively low standard deviation (less than 1.9). Figure 6c shows the graph measuring the lower limit of detection (LLOD) of the OFP sensor. As a result of performing ten experiments, the LLOD was 4 kPa, which is also indicated by the symbol “*” in Figure 6a. 

### 3.3. Omnidirectionality and Sensitivity Comparison between OFP and FSR Sensors

This section compares the performance of the proposed OFP sensor with that of FSP sensors in terms of omnidirectionality and sensitivity. FSR sensors are well known as one of the best commercialized technologies for measuring pressure with substantial sensitivity and measurable pressure range despite their low precision. [36,37,38,39,40,41,42].

**Figure 7:**
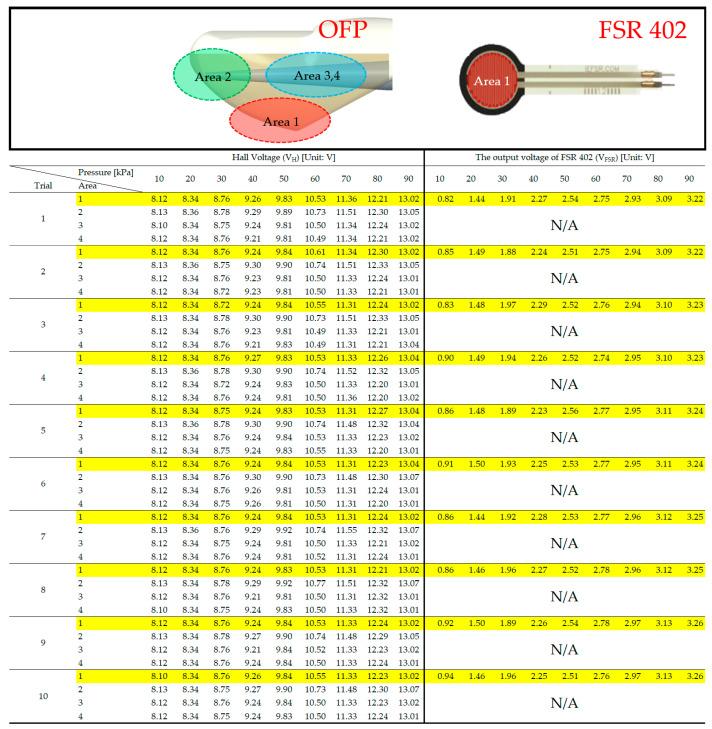
Experimental data measurements of OFP (**left**) and FSR402 sensor (**right**) over the general human touch pressure range.

We collected the experimental data, as shown in Figure 7, to quantify the reliability of the OFP sensor compared to that of the FSR402 sensor. Ten measurements were conducted under each pressure level ranging from LLOD to 90 kPa with a pressure level increment of 10 kPa. Based on the values reported in Figure 7, Table 1 calculates $\mu_{\sigma_{A}}$ and $\mu_{\sigma_{P}}$ to determine the consistency of the omnidirectionality of the OFP sensor, where the $\mu_{\sigma_{A}}$ indicates the average standard deviation of $\sigma_{V_{H}}$ for each pressure level over different areas, while the $\mu_{\sigma_{P}}$ is the average standard deviation of $\sigma_{V_{H}}$ over different pressures measured for a given area. 

As shown in Table 1, the $\mu_{\sigma_{A}}$ values are relatively small, between 0.024 and 0.227. This provides information on the consistency of how much consistently omnidirectional the OFP is, with the mean of $\mu_{\sigma_{A}}$ over different areas equal to 0.119. This result means that the OFP sensor has a strong linear relationship between pressure level and V_H_, regardless of the direction of pressure applied to the fingertip, thus having a high omnidirectionality. On the other hand, $\mu_{\sigma_{P}}$ tells us how reliable the OFP sensor is as a pressure sensor, where it shows the lowest and highest standard deviations: 0.118 and 0.128, with the mean of $\mu_{\sigma_{P}}$ over different pressure levels equal to 0.119. Therefore, we can conclude that our pressure sensor is omnidirectional enough while keeping the linearity of V_H_ with respect to pressure in all pressure directions.

**Figure 8:**
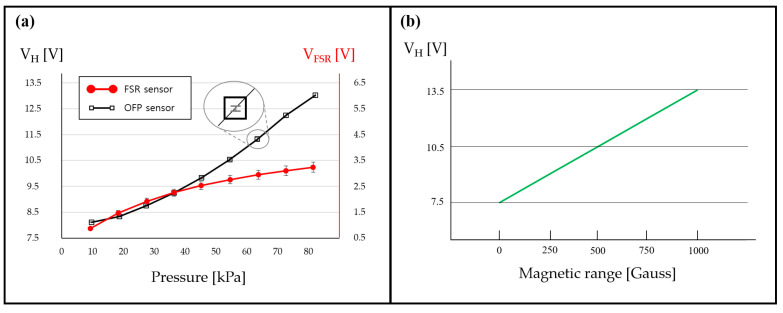
(**a**) Comparison of OFP and FSR402 sensors. The average relative standard deviation of the OFP sensor is less than 0.12, which is ten times less (better) than that of FSR402 (Measurement with Area 1). (**b**) Hall voltage vs. magnetic range [53,54].

Figure 8a shows that the FSR402 sensor has a good performance in terms of sensitivity and measurable pressure range. However, its precision is not sufficiently high. The issue of low precision has been documented as the main disadvantage of FSR402 sensors in several previous studies [49,50,51,52]. In contrast, our OFP sensor responds to pressures ranging from 4 to 90 kPa with a high sensitivity of 61.34 mV/kPa, which is 2 times better than that of FSR402 (32.80 mV/kPa). Furthermore, notice that the OFP has a much higher sensitivity than FSR402 for a pressure range greater than 50 kPa, below which they have approximately identical sensitivity levels. This outstanding performance significantly owed to the large available magnet slider displacement with respect to pressure changes, along with the remarkable linearity of the Hall sensor over a wide range of magnetic field strengths (0~1 KGauss) [53,54]. As shown in Figure 8b, the commercially available Hall sensor (Product name: SS49E) is linear enough over the target pressure range that we treat.

## 4. Conclusions

In this study, an omnidirectional fingertip pressure sensor was developed using the Hall effect. The sensor was integrated with an elastomer fingertip, Hall sensor, solenoid coil, and a movable magnet slider. The elastomer fingertip was designed to exhibit almost the same resilience as the skin of a human finger. The Hall sensor with the magnet slider was placed horizontally so that the Hall sensor can detect how closely the magnet slider approaches it while the slider horizontally moves in response to the external pressure. The solenoid coil was wrapped around the Hall sensor. It generates an electromagnetic force that makes it possible for the magnet slider to move back to the initial position. However, our pressure sensor still has some limitations. For example, when the external force is released, the magnet slider is supposed to move back to its initial position. But the problem is that we do not know exactly when the external force is released. Once we know the moment of the release, the magnet slider can be returned to the initial position by turning on the solenoid coil. Our future work includes (1) studying an automatic on-off solenoid mechanism that optically detects when the pressure is released, thus being able to automatically turn on or off the solenoid coil, and (2) exploring how the sensitivity is affected by the parameters, including material thickness in the fingertip, lubrication factor, surface smoothness of the slider, hardness of elastomer, humidity, temperature and so on. The experimental results show that our proposed sensor has a high sensitivity of up to 61.34 mV/kPa with a sensing range of up to 90 kPa, along with good linearity and omnidirectionality. Therefore, we are confident that our innovative fingertip pressure sensor using the Hall effect pioneers a new paradigm of pressure sensors and has a high potential for applications in robotics and biomimetic technology, as well as other research projects.

## Figures and Tables

**Table 1:** Calculation of $\mu_{\sigma_{A}}$ and $\mu_{\sigma_{P}}$ based on the OFP sensor experimental data from Figure 7, where the former is the average standard deviation of $\sigma_{V_{H}}$ for each pressure over different areas, while the latter is the average standard deviation of $\sigma_{V_{H}}$ over different pressures measured for a given area.

	10 kPa	20 kPa	30 kPa	40 kPa	50 kPa	60 kPa	70 kPa	80 kPa	90 kPa	$\mu_{\sigma_{P}}$
Area 1	0.058	0.000	0.166	0.113	0.079	0.223	0.137	0.227	0.056	0.118
Area 2	0.000	0.095	0.144	0.133	0.086	0.128	0.215	0.116	0.078	0.110
Area 3	0.058	0.000	0.167	0.158	0.145	0.138	0.075	0.266	0.061	0.119
Area 4	0.058	0.000	0.163	0.157	0.107	0.162	0.125	0.301	0.081	0.128
$\mu_{\sigma_{A}}$	0.044	0.024	0.160	0.140	0.104	0.163	0.138	0.227	0.069	

(Note: Each entry indicates the values of the standard deviation $\sigma_{V_{H}}$ of V_H_ for ten measurements with a given area and pressure level).

## Data Availability

Not applicable.
